# Mu rhythm desynchronization by tongue thrust observation

**DOI:** 10.3389/fnhum.2015.00501

**Published:** 2015-09-11

**Authors:** Kotoe Sakihara, Masumi Inagaki

**Affiliations:** ^1^Department of Developmental Disorders, National Institute of Mental Health, National Center of Neurology and PsychiatryKodaira, Japan; ^2^Department of Clinical Laboratory Science, Faculty of Medical Technology, Teikyo UniversityTokyo, Japan

**Keywords:** electroencephalogram, event-related desynchronization, event-related synchronization, tongue thrust observation, mu rhythm

## Abstract

We aimed to investigate the mu rhythm in the sensorimotor area during tongue thrust observation and to obtain an answer to the question as to how subtle non-verbal orofacial movement observation activates the sensorimotor area. Ten healthy volunteers performed finger tap execution, tongue thrust execution, and tongue thrust observation. The electroencephalogram (EEG) was recorded from 128 electrodes placed on the scalp, and regions of interest were set at sensorimotor areas. The event-related desynchronization (ERD) and event-related synchronization (ERS) for the mu rhythm (8–13 Hz) and beta (13−25 Hz) bands were measured. Tongue thrust observation induced mu rhythm ERD, and the ERD was detected at the left hemisphere regardless whether the observed tongue thrust was toward the left or right. Mu rhythm ERD was also recorded during tongue thrust execution. However, temporal analysis revealed that the ERD associated with tongue thrust observation preceded that associated with execution by approximately 2 s. Tongue thrust observation induces mu rhythm ERD in sensorimotor cortex with left hemispheric dominance.

## Introduction

In daily life, when acquaintances talk to us with their orofacial region masked, we often cannot judge whether they are speaking; hence, we delay in providing an appropriate response. This is because orofacial movement is one of the important cues for speech perception and conversation onset. There are two major explanation on how we perceive a speech (Sussman, 1989). The first is that speech perception is formed by sounds, and the second is the motor theory of speech perception. The first explanation is widely accepted, and it defines that the speech production and speech perception is separate processes. The second account maintains the idea that the ultimate components of speech are not sounds, but they are articulatory gestures; motor system intervenes in speech perception (Liberman and Whalen, 2000). The motor theory of speech perception has been reinforced by a series of experimental data obtained from the neurophysiology of the motor system. Studies conducted on monkeys showed that single neuron in the ventral premotor cortex discharge signals both when the monkey performs a specific action and when it observes an similar action (Gallese et al., 1996). This observation/execution matching system (di Pellegrino et al., 1992; Gallese et al., 1996; Rizzolatti et al., 1996) is named as the mirror neuron system, and the system converts observed action into one’s own action by forming a motor pattern replica in the brain. In addition, the mirror neuron system exists in the human premotor area and is involved in action recognition, understanding (Cochin et al., 1999; Muthukumaraswamy and Johnson, 2004a; Muthukumaraswamy et al., 2004; Oberman et al., 2005), and action imitation (Hari et al., 1998). The homology of the mechanism between the postulated motor theory of speech perception and the discovered observation/execution matching system (mirror neuron system) is obvious (Fadiga et al., 2002).

Anatomically, the primary motor areas are located downstream of the premotor area, and there are strong neuronal connections between premotor and primary motor areas (Greenlee et al., 2004). Then, mirror neurons in the premotor area activate the primary motor area. Moreover, human brain imaging (Buccino et al., 2001) and psychophysiological studies (Fadiga et al., 1995) have reported that action observation altered motor cortex excitability. When we see a hand, foot, or mouth movement performed by others, our corresponding hand, foot, and mouth regions of the primary sensorimotor area becomes active (Babiloni et al., 2002). The sensorimotor area activation owing to movement observation has been electrophysiologically studied since the 1950s (Gastaut and Bert, 1954; Chatrian et al., 1959) using a “mu rhythm” attenuation. A “mu rhythm” is a band between 8 and 13 Hz, and mu rhythm attenuation is also called as mu rhythm desynchronization. An oscillatory event-related desynchronization (ERD) is a response to specific external/internal events (Pfurtscheller and Lopes da Silva, 1999) and is associated with the activation of cortical neuron population (Pfurtscheller et al., 1997). Several previous studies have reported that both movement and movement observation induce the mu rhythm ERD in the primary motor cortex (Jurkiewicz et al., 2006; Muthukumaraswamy et al., 2006; Caetano et al., 2007), which reflects the activation (Gastaut and Bert, 1954; Chatrian et al., 1959; Bernier et al., 2007). Mu rhythm ERD in primary sensorimotor area is concomitant activation with mirror neuron activation by movement observation (Lopes da Silva, 2006).

Tongue thrust movement is a subtle orofacial movement. In Japanese speech, they do not show the tongue while pronouncing. According to the motor theory of speech perception postulate, speech perception depends on the observation of orofacial movement. The question of particular interest is as to how subtle orofacial movement is valid as a cue for speech perception. In other words, how subtle orofacial movement activates the sensorimotor area?

Tongue thrust movement activates sensorimotor cortex hemodynamically (Corfield et al., 1999; Fesl et al., 2003) and electrophysiologically (Nakasato et al., 2001). To date, there have been no studies that report the activation of the sensorimotor area by tongue thrust observation. In the present study, we used ERD as the cortical activation state and event-related synchronization (ERS) as the deactivation, inhibition, or at least the “idling” state (Pineda, 2005). We aimed to investigate the mu rhythm in the sensorimotor area during tongue thrust observation and obtain an answer to the question as to how subtle non-verbal orofacial movement observation activates the sensorimotor area.

## Materials and Methods

### Participants

Ten healthy adults (six males, 21−31 years old) participated in the present study. All participants were right-handed as assessed by the Edinburgh Handedness Inventory (Murias et al., 2007). None had histories of neurological or psychiatric illnesses. Each participant gave written informed consent to participate before experiment, but remained naive to the purpose of the study. The ethics committee at the National Center of Neurology and Psychiatry (NCNP) approved all experimental procedures.

### Recording

The scalp electroencephalogram (EEG) was acquired and recorded by a 128-channel Sensor Net system and Net Station 4.3.1. (Electrical Geodesic, Eugene, OR, USA; Muthukumaraswamy and Johnson, 2004b; Bernier et al., 2007; Streltsova et al., 2010). Electrode impedance was less than 50 kΩ. The EEG was sampled at 1,000 Hz without band-pass filtering. Raw EEG was recorded with the vertex (Cz) as the online reference and re-referenced off-line to the common average (Shimoyama et al., 2000). Motions were monitored and recorded by video camera for off-line analysis. The EEG recording system (Net Station 4.3.1.) synced EEG and motion picture. Based on the video data, trials in which participants moved inappropriately or made mistakes in performance were excluded.

### Procedures

**Figure 1:**
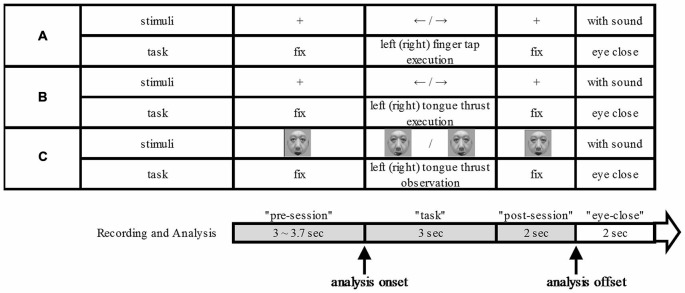
**Experimental scheme.** The three tasks: finger tap execution **(A)**, tongue thrust execution **(B)**, and tongue thrust observation **(C)**. For each task **(A–C)**, the upper column shows the visual stimulus presented on the monitor and the lower column the task performed. Each trial was divided into four temporal epochs: “pre-session”, “task” (beginning with stimulus presentation), “post-session”, and “eyes closed”. The analysis onset means in relation to visual cue.

Each participant sat on a comfortable chair 110 cm in front of a 17-inch Liquid Crystal Display (LCD). They kept their arms on the arm chair table and stayed as motionless and relaxed as possible. Each participant performed a finger tap execution, tongue thrust execution, and tongue thrust observation task as instructed by visual cues displayed on the LCD monitor (Figure 1). Presentation of visual cues was controlled by commercially available software (E-Prime 2.0. professional, Psychology Software Tools, Pittsburg, PA, USA). Each 10.0–10.7 s trial consisted of four phases: a 3.0−3.7-s “pre-session” rest epoch, a 3-s “task” epoch, a 2-s “post-session” rest epoch, and a 2-s “eyes closed” refresh epoch (Figure 1). We made the “eyes closed” refresh epoch to exclude the blink artifact during the “task” and “post-session” epochs. In the finger tap execution and tongue thrust execution tasks, a fixation cross, 4.4° × 4.7° in visual angle, was displayed at the center of the LCD monitor during “pre-session” and “post-session” epochs and an arrow with a visual angle of 7.3° × 3.1° was displayed during the “task” epoch. The fixation cross was also displayed at the same position during the “eyes closed” epoch while the music played. We played music during “eyes closed” refresh epoch to make the participants aware of the end time of the refresh epoch by stopping the music, after which they opened their eyes. The used music was a sound effect.

During the “pre-session” and “post-session”, participants remained at rest with eyes on the fixation cross. During the finger tap execution “task” epoch, the arrow pointed either right or left to indicate which index finger to tap. We instructed the participants to start and stop to tap as fast as they could by visual cue. In the tongue thrust execution task, the participant executed a brisk forward tongue thrust to either the right or left according to the arrow direction with as little articular movement as possible. We instructed the participants to start and stop tongue thrust as fast as they could by visual cue. Tongue thrust was followed by a return to the resting position. The jaw was kept relaxed and the mouth slightly open during the trial. During “eyes closed”, the participants closed their eyes while the music played.

The participants performed 40 trials each of right finger tapping, left finger tapping, right tongue thrust execution, and left tongue thrust execution tasks with the direction order randomized. During the “pre-session” and “post-session” epochs of the tongue thrust observation task, a still image of a male face (9.6° × 11.7° visual angle) was presented on the monitor. During the “task” epoch, participants watched a movie showing a tongue thrust to the right or left performed by an unfamiliar adult. We did not instruct the participants to associate the observed tongue thrust direction with that of their own tongue thrust. The movies of right and left tongue thrusts were presented a total of 40 times in random order (20 rightward and 20 leftward movements).

### ROI

**Figure 2:**
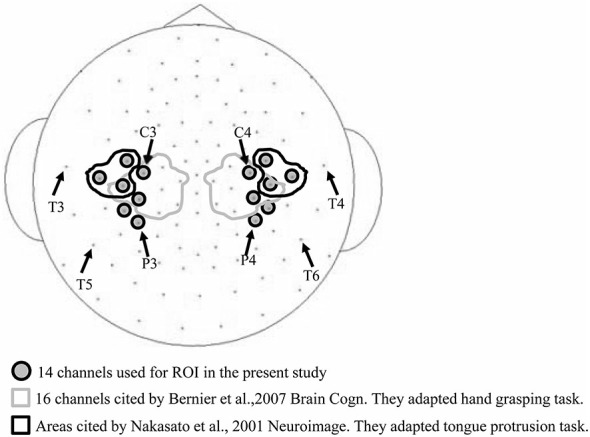
**Electrode channels used for the region of interest (ROI).** The 16 channels circled by the gray line were used to measure ERD/ERS in a hand grasping task (Bernier et al., 2007), and the six channels encircled by the black line in a tongue protrusion task (Nakasato et al., 2001). These arrays partially overlap with the 14 channels used in the current study (gray circles).

A previous study (Bernier et al., 2007) used a cluster of 16 electrodes surrounding the international 10–20 EEG electrode positions of C3 and C4 (demarcated by the gray lines in Figure 2) for assessing mu band ERD/ERS during a hand grasping and hand movement observation task. Another study Nakasato et al. (2001) measured the movement related magnetic fields (MRFs) associated with tongue protrusion using magnetoencephalogram (MEG), and the equivalent current dipole (ECD) was localized anterior and perpendicular to the central sulcus, areas bounded by the black line in Figure 2. Based on these studies, we chose a region of interest (ROI) of 14 bilateral electrode positions (gray in Figure 2) expected to cover hand and tongue motor areas. An electrocorticogram (ECoG) study found that tongue protrusion induced sustained alpha ERD in the left sensorimotor cortex (Crone et al., 1998) that varied considerably among individuals. Therefore, in the present study, we set a wide ROI to account for individual variability.

### Time-Frequency Analysis

Only trials in which participants correctly performed the task were used for time-frequency analysis. Trials with eye blinks and other movement artifacts were excluded by careful visual inspection of the raw EEG traces. We estimated the EEG power at each frequency as a function of time by continuous Morlet wavelet transformation in 0.5-Hz periods from 4–100 Hz using EMSE Suite v5.3 software (Source Signal Imaging, Inc. San Diego, CA, USA) as described (Moores et al., 2003; Muthukumaraswamy and Johnson, 2004b). The mu rhythm (8–13 Hz) and beta band (13−25 Hz) were analyzed. The frequency band of the mu rhythm was the same as the alpha band.

### Analysis of Event-Related Desynchronization or Synchronization (ERD/ERS)

To estimate ERD and ERS, the EEG power spectrum was first divided into 200-ms time windows. Starting at task onset, we calculated the EEG power ratio of each successive 200-ms epoch relative to baseline, defined as the last 200-ms of the “pre-session” (Figure 1). Finally, ratios were log-transformed (Bernier et al., 2007) to account for individual variability and non-normality of the distribution. A positive score (enhanced EEG power) indicates ERS while a negative score (diminished EEG power) indicates ERD.

### Statistical Analysis

In a previous finger tap study, the ERD lasted until approximately 1 s after movement stopped (Babiloni et al., 2002). Thus, in the finger tap, tongue thrust execution, and tongue thrust observation tasks used here (Figure 1), the 2-s “post-session” was divided into the former 1 s as the “post-session 1” and the latter 1 s as the “post-session 2.” Subsequently, statistical significance was calculated among four epochs and the last 1.8 s of the “pre-session”, “task”, “post-session 1”, and “post-session 2” using independent samples Kruskal-Wallis tests with multiple comparison correction. Additionally, we compared the temporal shift in ERD from “task” with “post-session” in the finger tap execution, tongue thrust execution, and tongue thrust observation tasks. For this purpose, we divided the 5-s “task” plus “post-session” epochs into successive 0.8-ms time windows. The EEG values of the five time windows were compared with those of the “pre-session” 1.8 s baseline. *T*-tests with Bonferroni correction were used for statistical analysis. All statistical analyses were performed using IBM SPSS Statistics software (version 20-J).

## Results

### Finger Tap Execution

**Figure 3:**
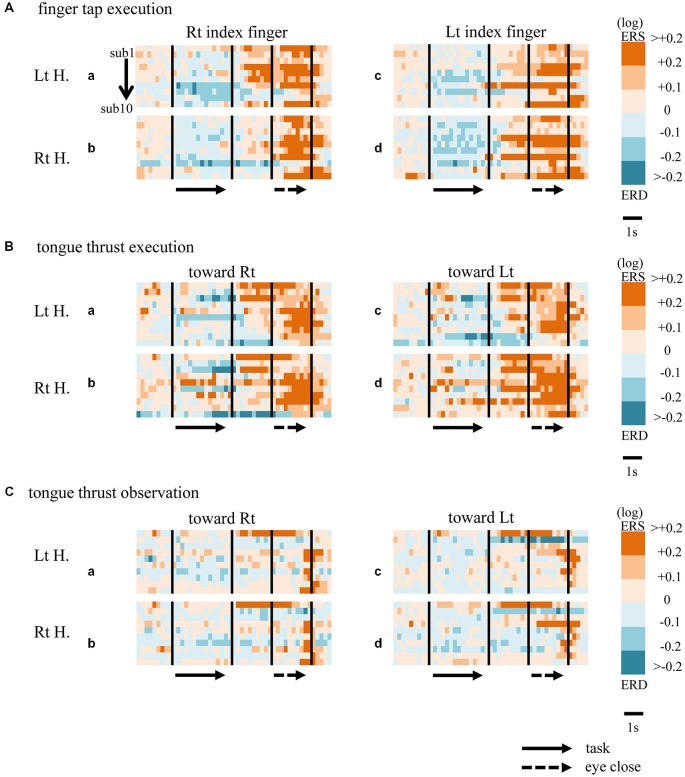
**Matrix chart of beta band ERD/ERS with time (horizontal scale) from all ten participants (sub1−sub10, arranged vertically).** Light and deep blue indicates ERD, orange and red ERS. Matrices are for finger tap execution **(A)**, tongue thrust execution **(B)**, and tongue thrust observation **(C)**. H, hemisphere; Lt, left; Rt, right.

**Figure 4:**
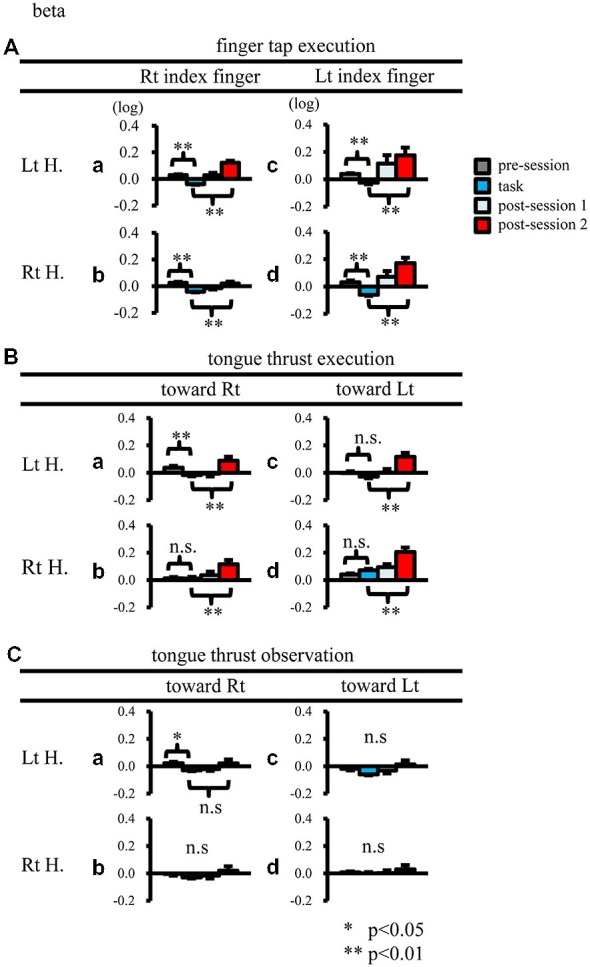
**Logarithmic electroencephalogram (EEG) power ratios (ERD, negative; ERS, positive) for the beta band.** Upper columns are log (ratios) for finger tap execution **(A)**, middle for tongue thrust execution **(B)**, and bottom for tongue thrust observation **(C)**. Each plot **(a–d)** represents the log (ratio) during each epoch (pre-session, task, post-session 1, post-session 2) in the left hemisphere (Lt H., **a** and **c**) and right hemisphere (Rt H., **b** and **d**) evoked by right index finger tapping **(A-a,b)**, rightward tongue thrust execution **(B-a,b)**, rightward tongue thrust observation **(C-a,b)**, left index finger tapping **(A-c,d)**, leftward tongue thrust execution **(B-c,d)**, and leftward tongue thrust observation **(C-c,d)**. ^*^p < 0.05; ^**^p < 0.01.

**Figure 5:**
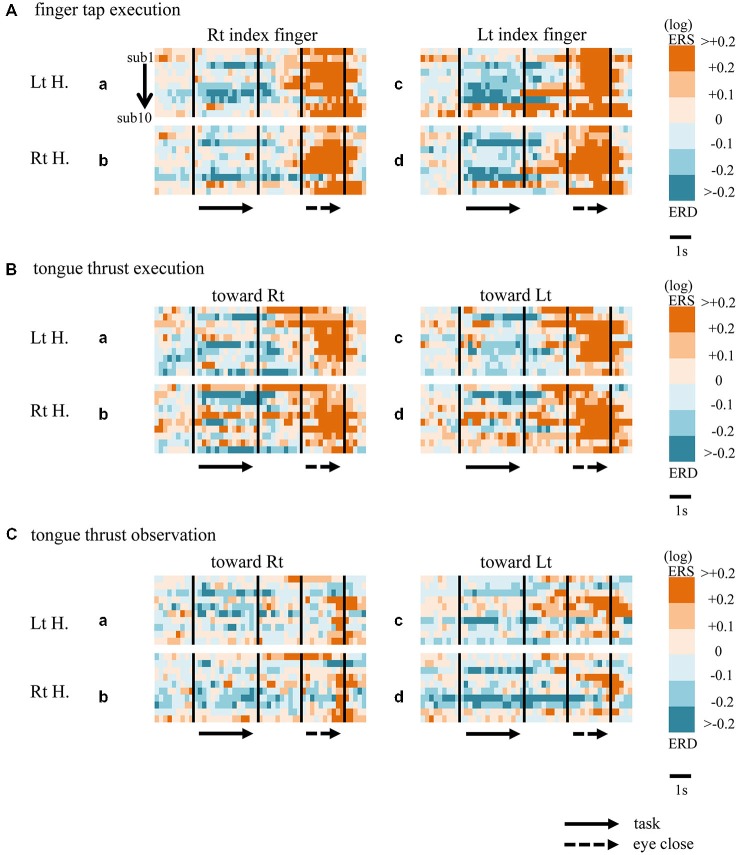
**Matrix chart of mu rhythm ERD/ERS from all ten participants (sub1−sub10).** ERD indicated by light/deep blue, ERS by orange/red. Matrices are for finger tap execution **(A)**, tongue thrust execution **(B)**, and tongue thrust observation **(C)**. H, hemisphere; Lt, left; Rt, right.

**Figure 6:**
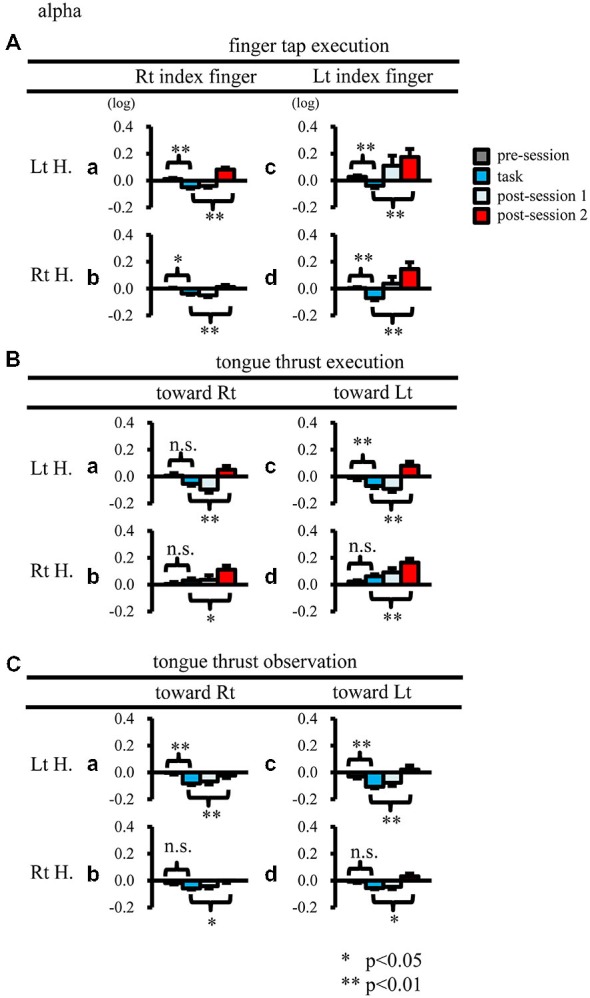
**Logarithmic EEG power ratios (ERD negative; ERS positive) for the mu rhythm.** The plot organization is as that mentioned in Figure 4. H, hemisphere; Lt, left; Rt, right. ^*^p < 0.05; ^**^p < 0.01.

When participants performed finger taps using their right index finger, a decrease in beta band power was seen predominantly in the left hemisphere (Figure 3A-a), and the decrease during the “task” phase was significant relative to the “pre-session” baseline (Figure 4A-a, *p* < 0.01). Beta band ERD was shown during “post-session 1”, and beta band ERS was shown during “post-session 2” (Figure 3A-a). The increase in beta band power (ERS) during “post-session 2” was significant relative to the “task” epoch (Figure 4A-a, *p* < 0.01). Strong beta band and mu rhythm ERS during the “eyes closed” refresh epoch was determined to be an eye blink artifact based on examination of raw EEG traces. When participants performed finger tapping using their left index finger, a similar temporal sequence of beta band ERD followed by ERS was seen in the right hemisphere (Figure 3A-d), and both changes were significant (Figure 4A(a–d); *p* < 0.01). Like the beta band, the mu rhythm also showed a sequential ERD/ERS evoked by finger tapping (Figures 5A-a,d, 6A-a,d; *p* < 0.01).

### Tongue Thrust Execution

When participants performed tongue thrust execution toward the right, beta band ERD appeared in the left hemisphere during the “task” epoch relative to the “pre-session” baseline (Figures 3B-a, 4B-a; *p* < 0.01), while no significant ERD was observed in the right hemisphere. During “post-session 2”, beta band power increased in both hemispheres, resulting in bilateral ERS (Figures 3B-a, 4B-a,b; *p* < 0.01 vs. the task phase). Mu rhythm power showed a decrease in the left hemisphere when participants performed tongue thrust execution toward the left (Figure 5B-c), and this mu rhythm ERD during the “task” epoch was significant compared with the “pre-session” (Figure 6B-c; *p* < 0.01). During “post-session 2”, mu rhythm ERS appeared (Figure 5B-c) that was significant compared with the “task” phase (Figure 6B-c; *p* < 0.01). This mu band ERS demonstrated left hemispheric whether tongue thrust execution was toward the right or toward the left.

**Figure 7:**
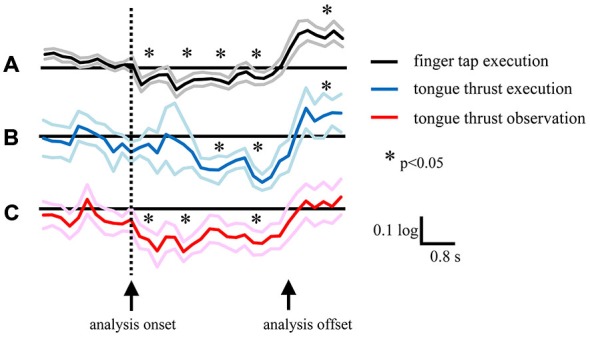
**Grand average of logarithmic EEG power ratios for the beta band in the left hemisphere during right finger tap execution (A), mu band during leftward tongue thrust execution (B), and mu band during leftward tongue thrust observation (C).** Black, blue, and red lines indicate mean. Gray, light blue, and pink lines indicate plus or minus one standard error. ^*^p < 0.05.

There was a significant time shift in the appearance of EEG desynchronization between the finger tap and tongue thrust tasks (Figures 7A,B). The grand average mu power ratio during the tongue thrust execution “task” phase showed significant desynchronization (mu ERD) 1.6–2.4 s after visual cue presentation (the direction arrow; Figure 7B), while significant beta band desynchronization (beta ERD) in the finger tap execution task was observed 0−0.8 s after visual cue presentation (Figure 7A). Tongue thrust execution evoked significantly greater ERD compared with finger tap execution at 1.6–2.4 s, 2.4–3.2 s, and 3.2–4.0 s relative to cue presentation.

### Tongue Thrust Observation

Significant mu rhythm ERD was also seen in the left hemisphere while observing another person’s tongue thrust execution toward either right or left compared with pre-session baseline (Figures 5C-a,c, 6C-a,c; *p* < 0.01). This ERD reversed to ERS during “post-session 2”, (Figure 5C-a,c), with a significant difference compared with the “task” phase (Figure 6C-a,c; *p* < 0.01). In contrast to mu activity, the beta band showed slightly significant ERD during the observation (“task” phase) and no significant ERS during “post-session 2” (Figures 3C, 4C).

### Temporal Shift in ERD During Tongue Thrust Observation vs. Execution

In the tongue thrust observation task, the mu power showed significant desynchronization at 0−0.8 s after visual cue presentation (Figure 7C), significantly earlier than during tongue thrust execution (Figure 7B) and more akin to the beta ERD during the finger tap execution task (Figure 7A). The mu power showed significantly greater ERD in the tongue thrust execution task at 1.6–2.4 s, 2.4–3.2 s, and 3.2–4.0 s after cue presentation compared with the observation task.

## Discussion

The three critical results of this study are that: (1) tongue thrust observation induced mu rhythm desynchronization; (2) tongue thrust observation toward either left or right dominantly induced mu rhythm desynchronization with left hemisphere; and (3) mu rhythm desynchronization induced by tongue thrust observation preceded that evoked by tongue thrust execution by approximately 2 s.

### Finger Tap Execution

Both mu rhythm and beta band ERD appeared in bilateral sensorimotor areas during finger tap execution. A simple finger movement with self-pacing has been shown to induce mu rhythm ERD in bilateral sensorimotor cortex by MEG (Salmelin et al., 1995). Both mu rhythm and beta band ERD in the contralateral sensorimotor area contributes to motor planning before movement, while ERD in the bilateral sensorimotor areas during movement contributes to motor execution (Pfurtscheller, 1989; Crone et al., 1998). In the present study, the “task” time window beginning at the onset of a visual cue included cortical activities associated with both motor planning and execution. Beta ERS reproducibly appeared after finger tap execution in the present study. Beta ERS follows beta ERD after the movement stops (Neuper and Pfurtscheller, 2001), and it is termed as “beta rebound” (Cassim et al., 2001). A TMS study indicated that beta rebound reflects the decline of motor cortex excitability (Chen et al., 1998). This beta rebound continued for approximately 1 s after movement stopped. It was suggested play a role in suppression so that this maintained ERS reflects return of cortical brain activity to resting levels. Moreover, others have reported that beta rebound may reflect not only simple suppression but also an active process, as the beta rebound was facilitated when movement was voluntarily stopped (Alegre et al., 2008) but not when passively stopped. While the mechanism and function of beta rebound remain controversial, the present study suggests that beta ERS suppresses cortical activity (or reflects suppression) after movement has already stopped.

### Tongue Thrust Execution

Beta band ERD evoked by tongue thrust execution was dominantly showed at the left hemisphere (Figure 4B). However, the tongue thrusts used in previous studies (Corfield et al., 1999; Fesl et al., 2003) differed from those used here, possibly accounting for this inconsistency. Previous reports have shown that tongue contraction activates bilateral sensorimotor cortex (S1/M1; Corfield et al., 1999), and that tongue movements from the right to the left activate the bilateral tongue motor area (Fesl et al., 2003).

Mu rhythm ERS was induced in the left hemisphere by tongue thrust execution toward the left (Figure 6B-c). Like mu rhythm ERS evoked by finger tap execution, mu rhythm ERS evoked by tongue thrust execution is thought to reflect inhibitory activity. Mu rhythm ERS was induced in the hand motor area by tongue movement (Pfurtscheller and Neuper, 1994), where it was proposed that the excitation of one primary sensorimotor area was accompanied by the inhibition of other sensorimotor areas. The ROI in the present study included both hand and tongue sensorimotor areas; hence, mu rhythm ERS may reflect both suppression of the tongue motor area itself and suppression of hand motor area.

### Tongue Thrust Observation

To our knowledge, this is the first study to report mu rhythm ERD in the sensorimotor cortex induced by tongue thrust observation. There are two possibilities to explain this mu rhythm ERD in the sensorimotor area. One possibility is the mirror neuron. The mu rhythm ERD in the sensorimotor area during extremity observation has been considered by the mirror neuron activation in the premotor area (Jurkiewicz et al., 2006; Lopes da Silva, 2006; Muthukumaraswamy et al., 2006; Caetano et al., 2007). According to these previous findings, in the present study, we speculate that mu rhythm ERD may be caused of mirror neuron activation. The other possibility is visual motion itself. Mu rhythm ERD was found in the sensorimotor area during the observation of non-biological motion such as opening and closing of the aperture (Muthukumaraswamy et al., 2006). They considered that participants internally projected their mouths onto the aperture, and suggested that the sensorimotor area is involved in the perception of structural event sequences. In addition, brushing motion only induces the mu rhythm ERD in the sensorimotor area (Cheyne et al., 2003). This activation is interpreted to be related to transient visual stimulation that is supported by reports of automatic activation of primary motor area by various forms of visual input (Koshino and Niedermeyer, 1975; Vanni et al., 1999). During the observation of brush movement, the participants could expect that the brush movement was the result of a particular action performed in the experiment, and they discussed that the observation of tool movement that enables an individual to associate it with one’s own action is some form of action observation (Cheyne et al., 2003). In the present study, we need to consider that mu rhythm ERD in the sensorimotor areas may be a non-specific effect in terms of the visual input. Because we did not include a control condition that is visual motion alone, we needed to perform another study to clearly eliminate this possibility.

Mu rhythm ERD evoked by tongue thrust observation showed left hemispheric dominance. One explanation is that tongue thrust is an orofacial movement associated with speech, which may activate the left IFG via nearby Broca’s area. Another possible explanation is that mirror neurons are more likely to be formed within the left IFG of these right-handed participants. Heyes (2010) proposed that the mirror neuron is a byproduct of associative learning; that is, motor neurons become mirror neurons through the sensorimotor experience of observing and executing the same action, termed the “associative hypothesis” (Heyes, 2010). Based on the associative hypothesis, participants with left hemispheric dominance of tongue thrust execution would more likely develop mirror neurons in the left hemisphere.

### Differences in ERD Magnitude Between Tongue Thrust Execution and Observation

In the present study, the mu rhythm ERD did not differ between tongue thrust execution and observation. In a previous finger extension task study, however, the mu rhythm showed significantly greater ERD during execution compared with observation (Babiloni et al., 2002). Alternatively, the mu rhythm ERD during hand grasping did not differ between execution and observation (Bernier et al., 2007). Thus, differences in the magnitude of the ERD between execution and observation may depend on the specific action.

### Temporal Shift of EEG Power

#### Finger Tap Execution vs. Tongue Thrust Execution

Mu rhythm ERD evoked by tongue thrust execution occurred approximately at 2 s later than the occurrence of the beta ERD evoked by finger tap execution (Figures 7A,B). The Bereitschafts potential (BP) is a movement-related cortical potential in which the onset time reflects the readiness time to perform a movement (Ikeda et al., 1995), and the BP onset reflects the complexity of the movement (Satow et al., 2004). The mean BP onset of tongue protrusion was 1.74 ± 0.23 s (Satow et al., 2004) longer than that of right finger extension (1.69 ± 0.24 s) or left finger extension (1.56 ± 0.24 s; Barrett et al., 1986). The BP onset of tongue protrusion is slightly longer because it is a more complex movement and so requires more motor preparation time. In our study, the longer time delay of mu rhythm ERD during tongue thrust execution trials may also reflect movement complexity compared with finger tapping. The other probable reason of delayed mu ERD is the slower onset and longer duration of the tongue movement.

#### Tongue Thrust Execution vs. Tongue Thrust Observation

Mu rhythm ERD evoked by tongue thrust observation was induced approximately at 2 s earlier than that evoked by tongue thrust execution. There are three possible reasons for this temporal shift. The first reason is that, in tongue thrust observation, visual information on movement directly activates mirror neurons, while in tongue thrust execution, the visual cue is translated to a motor command without mirror neuron activation. The second reason is that a previous study reported that the peak latency of mu rhythm ERD during finger tap extension was shorter than for finger tap observation (562 ± 71 ms vs. 656 ± 74; Babiloni et al., 2002) although the difference did not reach statistical significance. In contrast, we found a marked difference, possible due to the additional complexity of tongue thrust, which takes more time to perform. The third reason is the analysis onset of tongue movement. Because it is difficult to record an electromyography of the tongue thrust and achieve the right start time of the movement, we analyzed the EEG during movement by a visual cue on the display. The onset difference between movement and observation may provide an effect to the ERD temporal shift, and this is the limitation of our study.

## Conflict of Interest Statement

The authors declare that the research was conducted in the absence of any commercial or financial relationships that could be construed as a potential conflict of interest.
